# Evaluation and perceptions of a nursing discharge plan among nurses from different healthcare settings in Spain

**DOI:** 10.1186/s12913-022-08109-9

**Published:** 2022-05-28

**Authors:** Gloria Reig-Garcia, Anna Bonmatí-Tomàs, Rosa Suñer-Soler, Mari Carmen Malagón-Aguilera, Sandra Gelabert-Vilella, Cristina Bosch-Farré, Susana Mantas-Jimenez, Dolors Juvinyà-Canal

**Affiliations:** 1 Department of Nursing, University of Girona, Emili Grahït, 77 17003 Girona, Spain; 2 Health and Healthcare Research Group, University of Girona, Girona, Spain

**Keywords:** Discharge planning, nursing discharge plan, continuity of care, Quality of care, Nurse management

## Abstract

**Purpose:**

The exchange of information between different healthcare settings through a nursing discharge plan is essential for safe care. However, the factors contributing to achieving the most efficient exchange have not been well studied. This study aimed to evaluate and explore the perceptions of a nursing discharge plan from the perspective of nurses in different healthcare settings.

**Methods:**

A mixed methods approach comprising a specifically designed ad hoc questionnaire (*n* = 437) and a focus group session (*n* = 8).

**Findings:**

Overall, 66.1% out of 437 nurses, and especially those working in nursing homes, were satisfied with the nursing discharge plan.

Lack of time to complete the report and poor information about both nursing diagnoses and patients’ social assessment were identified as problem areas. Some proposals emerged from the focus group: providing sufficient time for its completion, giving the nursing discharge plan a more flexible structure permitting more open-ended responses, requiring more information to be provided about the social and psychological situation of the patients, training nurses to use standardized language to avoid possible misinterpretations, and getting nurses from the different health care settings to work together in designing continuity of care plans. Elderly and low-income patients are found to need greater attention when filling out nursing discharge plans.

**Conclusions:**

The study has revealed key aspects that need to be improved and some recommendations in implementing the nursing discharge plan in our health area. These include that there should be more time provided to complete the NDP, and also specific details regarding the format, structure, content of the information that is communicated, and the prioritization of the patient profile.

**Supplementary Information:**

The online version contains supplementary material available at 10.1186/s12913-022-08109-9.

## Background

Discharge planning is a dynamic and systematic care process beginning when a person is admitted to hospital that has the main objective of preparing the patient and family to maintain their functional capacity and well-being after hospitalization [1]. This planning could reduce readmissions 3 months after discharge [2]. Numerous organizational factors (shortage of staff nurses and lack of discharge planning policies), personal factors (weak communication skills and poor patient understanding of the discharge process), and sociocultural factors (insufficient social support) can negatively influence the discharge process [3, 4]. Nowadays, standardized discharge plans are considered to be an important initiative in improving this process [5]. However, it has been noted that some aspects of the discharge process still need to be properly studied [6].

A nursing discharge plan (NDP) should include, in a clear, structured and summarized way, all the processes carried out during the hospitalization, focusing on the biological, psychological and social situation of the patient and family [7]. A useful NDP facilitates the accurate transmission of information and continuity of care between the different areas of care [8, 9]. It also improves the detection of risk situations early on and avoids errors [10, 11]. In addition, the NDP can favor a reduction in the length of hospital stays and readmission rates if it is planned before the day of discharge [12].

The information exchange (i.e. continuity of information) between different care settings during hospital discharge is essential for safe care. In this respect, national standards have been introduced in recent years in some countries with the aim to improving and standardizing the information that needs to be communicated [12–14]. Such standards can improve the quality of communication at discharge, but audits have shown adherence to these recommendations to be variable [15]. Additionally, some studies have shown a lack of standardized routines and structures to convey the information and that sometimes this information is unclear [16, 17]. Few studies report barriers to discharge planning or initiatives to improve it [18, 19].

### Nursing discharge plan of Girona

A working group made up of management and nurses from different care settings agreed on a common NDP for use across the whole province. The NDP, which was designed based on empirical knowledge, has an electronic format and a rigid and standardized structure. According to Bunkenborg [20], standardizing the discharge plan structure is a key factor in addressing patients’ transition to different care settings. Moreover, electronic records not only provide greater organizational efficiency but also reduce the risk of errors in caring for patients [21]. The NDP is structured in three dimensions. The first part, Diagnosis and Summary of Hospital Admission, records socio-demographic data, the main diagnoses at admission, medication, allergies, usual diet and any specific nutritional requirements. The second part, Physical and Social Assessment of the Patient at Hospital Discharge, includes the evaluation of autonomy for daily living activities (hygiene, preparing food, getting dressed, toilet use, etc.) and the Barthel Index score [22], and vital signs (temperature, blood pressure, pulse oximetry, and heart rate). This part also includes information related to the knowledge and skills of the caregiver in caring for the patient (familiarity with the types of cures, treatments prescribed, etc.). Finally, the third part, Care Plan and Control Schedule, records the treatment plan on discharge (pharmacological, non-pharmacological and cures) as well as information about follow-up visits at the hospital.

Interest has recently been given to increasing the quality of nursing documentation in the healthcare sectors [23]. Assessing the quality of NDPs could provide insight into the best practices and limitations to improve both their quality and the quality of patient outcomes. However, most studies into NDPs focus on patients’ subjective experiences [18] and few on the knowledge, perceptions and practices of the professionals involved [24]. In the Girona health region (Spain), an electronic standardized nursing discharge plan was developed in order to improve the quality of the continuity of information. The present study aimed to evaluate and explore the different perspectives on this NDP among nurses from a variety of workplace settings (hospitals, primary care centers and nursing homes).

## Methods

The present study used a mixed methods approach [25] with an explanatory sequential design [26], consisting of a sequential triangulation with a first phase of quantitative data collection and analysis through the use of a specifically designed ad hoc questionnaire followed by the collection of qualitative data through the use of a focus group to describe different perspectives of our health area’s NDP [27].

### Quantitative research

A total of 21 public healthcare centers were included in this study. These were all the primary care centers (*n* = 13) and hospitals (*n* = 2) of the Girona region, and a convenience sample of six nursing home settings. All the nurses from the different care settings (*n* = 639) were invited by the researchers to self-complete a short anonymous ad hoc questionnaire (see Additional file 1: Annex 1). The questionnaire consisted of two different parts. The first part collected sociodemographic and work-related information of the participants, such as age, sex, years since graduation, work experience in the present position, employment relationship, function, training, and research activities. The second part focused on the participants’ perception of the standardized NDP and consisted of four questions: (a) satisfaction, (b) review and use (c) time to devote to the NDP, and (d) assessment of the contents related to the following items: diagnoses at discharge, summary of the hospital admission, patient assessment at discharge, care plan, recommendations at discharge, and forthcoming controls.

Questions a), b) and c) were measured with a five level Likert scale whereas question d), which required a more specific evaluation, was measured with a ten-point scale. Both clinical and methodological experts participated in the questionnaire’s preparation.

A pilot questionnaire was given to 18 nurses working in different care levels, resulting in minor modifications being made. The internal consistency reliability was examined by calculating Cronbach’s alpha. Alpha values were all > 0.8, indicating that the reliability of the scales was good.

Continuous variables were described as the mean and measures of dispersion (standard deviation, median, and interquartile range). Categorical variables were described in terms of absolute frequency and percentage. Anova was used to compare continuous variables with categorical variables. The chi-squared test was used to compare categorical variables.

The data obtained from the questionnaires were analyzed using IBM SPSS Statistics for Windows v. 21.0 (IBM Corp. Released, 2012). The level of significance for all analyses was set at *p* < 0.05.

### Qualitative research

A generic qualitative design [28] based on a constructivist naturalistic approach was adopted. A qualitative methodology offers the possibility of understanding the complexity of a phenomenon from the differing points of view of informants [29]. Generic studies offer an opportunity for researchers to play with boundaries, use the tools provided by established methodologies, and develop research designs that fit their epistemological stance, discipline, and particular research questions [30].

The method of the research presented here consisted of a focus group session with eight nurses. These were recruited 4 months after the analysis of the quantitative data. Participants were selected from all the different health care settings using an intentional sample [31]. The homogeneity criterion for the sample selection was the ability of the participant to provide relevant information, and the heterogeneity criteria were the workplace care setting, the job function and the work experience.

A semi-structured guide based on the quantitative research results was used with open-ended questions related to the nursing discharge plan [32] (Table 1). The focus group was moderated by one of the members of the research team while another researcher of the team took field notes during the session. The notes included aspects of non-verbal communication and summaries of the participants’ discussion that were used to report back at the end of the session. The focus group session lasted 60 minutes and took place on a hospital ward. The session was audio-recorded and transcribed verbatim. The qualitative data were analyzed by two different researchers using content analysis. Krippendorff [33] defined content analysis as “a research technique for making replicable and valid inferences from texts (or other meaningful matter) to the contexts of their use”. The process followed in conducting qualitative content analysis is composed of four stages: decontextualization, recontextualization, categorization and compilation [34]. To increase the validity of all the results, the themes were discussed and clarified until a consensus was reached [35].

**Table 1:** Questions used to generate the focus group discussion

1. Explain your level of satisfaction regarding the NDP.	
2. In your opinion, what would the ideal structure of an NDP be like?	
3. In your opinion, what is the most important information regarding the continuity of patient care?	
4. From your viewpoint, does the current NDP miss important information? What should be added?	
5. What difficulties do you encounter when completing or reviewing the NDP?	
6. What type of patient profiles do you think have a greater need for continuity of care?	
7. What improvements would you propose to be made to the current NDP?	

### Ethical considerations

This study was conducted in accordance with the principles of the Declaration of Helsinki. The study was carried out in compliance with all relevant regulations and guidelines and was approved by the management boards of the Institut Català de la Salut and the Institut d’Assistència Sanitària of the Girona health region. Given that the study only consisted of an evaluation of the NDP by nurses as part of a process of continuous improvement in care quality, it was not necessary to submit the study for approval by the local ethics committee. Participation was completely voluntary and informed consent was given by all the participants.

## Results

### Results of the quantitative research: nurses’ assessment of the NDP

A total of 437 out of 639 nurses completed the questionnaire with response rates ranging from 68.96% (hospital nurses) to 77.27% (nursing home nurses). The sociodemographic and occupational characteristics of the participants are shown in Table 2. 66.1% of the nurses were highly satisfied with the NDP. Having a permanent employment relationship (69.9%, *p* < 0.05), and working in a nursing home (97.2%, *p* < 0.00) were associated with greater satisfaction with the NDP. Primary care nurses were the least satisfied (Table 3). 89.9% of the nurses stated that they reviewed and took into account all the NDPs they received. More experienced (14.73%, *p* < 0.00), older (44.18%, *p* < 0.00), and postgraduate (95.3%, *p* < 0.00) nurses stated that they used the NDP more. The perception of lack of time to perform or review the reports was present in 71.7% of the nurses, especially those with a temporary employment relationship (85.7%, *p* < 0.00), working in a nursing home (82.4%, *p* < 0.00), and those without a postgraduate (82.6%, *p* < 0.05) or PhD (72%, *p* < 0.05) education.

**Table 2:** Sociodemographic and occupational characteristics of the sample

	Total study population (N:437)
**Age (mean; SD)**	40.5 (10.7)
**Years after completing university studies (mean; SD)**	18.1 (10.7)
**Years of work experience in the same care level (mean; SD)**	14.9 (10.1)
**Sex (n; %)**	
Women	404 (92.4)
**Employment relationship (Contract) (n; %)**	
Permanent	302 (69.1)
Temporary	135 (30.9)
**Function (n; %)**	
Care	408 (93.4)
Management	22 (5.1)
Liaison nurse or case management nurse	7 (1.5)
**Training (n; %)**	
Continuous	354 (81.2)
Postgraduate	316 (72.4)
**Research (n; %)**	
Publications in the last 5 years	76 (17.6)
Attended congresses/activities on continuity of care	199 (45.7)

**Table 3:** Nurses’ level of satisfaction with the nursing discharge plan and related variables

	Nurses’ satisfaction regarding nursing discharge plan			
	High (1–2) *n* = 289	Medium (3) *n* = 119	Low (4–5) *n* = 29	p
**Age (mean; SD)**	40.5 (10.6)	40.9 (11.2)	39.6 (10.1)	0.94^a^
**Years since completing university studies (mean; SD)**	18.2 (10.5)	17.9 (11.3)	17.1 (10.8)	0.83^a^
**Years working (mean; SD)**	15.2 (10.3)	14.3 (9.9)	13.9 (8.9)	0.68^a^
**Employment relationship (n; %)**				
Permanent contract	211 (69.9)	67 (22.2)	24 (7.9)	0.03^b*^
Temporary contract	78 (57.8)	52 (38.5)	5 (3.7)	
**Care setting (n; %)**				
Primary care	92 (60.1)	43 (28.1)	18 (11.8)	0.01^b*^
Hospital	171 (68.7)	71 (28.5)	7 (2.8)	
Nursing home	26 (76.5)	5 (14.7)	3 (8.8)	
**Functions (n; %)**				
Care	260 (65.2)	112 (28.1)	27 (6.8)	0.43^b^
Management	18 (81.8)	4 (18.2)	0 (0.0)	
Liaison nurse or case management nurse	4 (57.1)	2 (28.6)	1 (14.3)	

The continuous variables are described with the mean and standard deviation and the categorical variables with the absolute frequency and their percentage

*p<0.05 is considered significant

^a^Anova was used to compare continuous variables with categorical variables

^b^The chi-squared test was used to compare categorical variables

**Fig. 1:**
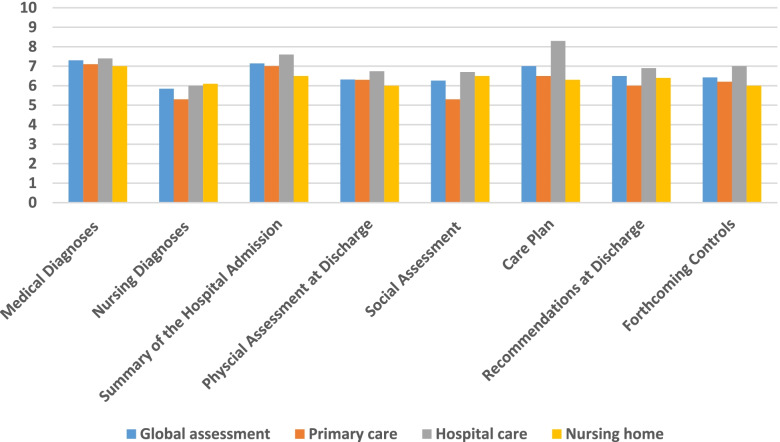
Level of satisfaction related to the different sections of the Girona Nursing Discharge Plan

Satisfaction related to the different sections of the NDP is shown in Fig. 1. All sections received scores above 5 (range 0–10). The section on medical diagnoses received the highest score (7.35; 2.9), while the section on nursing diagnoses (5.84; 3.3) and social assessment of the patient at discharge (6.26; 2.8) received the lowest scores. However, nurses with a permanent employment relationship (6.41(3.4); *p* < 0.05) and with research training (7.25 (2.9); *p* < 0.01) rated the nursing diagnoses of the NDP with higher scores. Hospital nurses (6.74 (2.6), *p* < 0.00) rated the content related to the patient’s physical assessment at discharge and to the care plan more positively (8.34 (1.3), *p* < 0.00).

### Results of the focus group analysis

A focus group was conducted with eight nurses. The age of the participants ranged from 26 to 56 years. Six nurses worked directly with care (2 from the hospital, 3 from primary care and 1 from the nursing home), and 2 were nurse managers. The analysis revealed three themes related to the NDP: (1) perception of the structure, format, and completion; (2) perception of the contents; and (3) perception of which patients had a greater need for discharge planning.

### Nurses’ perceptions of the structure, format, and completion

Overall nurses were satisfied with the standardized structure of the NDP as it guaranteed a certain homogeneity in the communication of the pertinent information and, in so doing, ensured appropriate continuity in the care that patients receive.

“...without a structure we would surely leave out many aspects that need to be communicated” [P7].

However, they acknowledged that they sometimes completed sections of the report automatically, without contrasting the information with the real situation of the patient. Primary care nurses particularly noticed this when they read reports and then visited patients.

“I think we have become so used to ticking boxes when filling in the form, and sometimes we do it so automatically that we do not say what is really important for other nurses.” [P4].

“You notice that some of the information that is filled in has not been properly verified against the patient’s real situation.” [P2].

Primary care nurses suggested that it could be improved by having an open-ended section that would facilitate the communication of aspects not included in the structured elements of the reports.

“The report should have an open space so that we can write down whatever we consider necessary.” [P3].

With regards to the format, emphasis was placed on the use of standard nursing language and the avoidance of abbreviations in the free text of reports.

“Not all of us use the same nursing language...and this needs to be improved...sometimes abbreviations are used and then what is being said is not understood correctly.” [P7].

With regards to the completion of the report, the nurses pointed out the importance of starting the NDP well in advance of the discharge date.

“The report needs to be worked on before the discharge day [P2]”.

However, they acknowledged that they frequently complete the NDP just before the patient’s discharge, mostly due to lack of time.

“It’s a time when you need to sit down and concentrate, and there are always other things to do before that.” [P1].

The nurses pointed to the need for tools to facilitate the co-editing of reports such as having a shared space for the nurses who have attended the patient during the hospitalization.

“...it would be very good if we had a space where we could comment on what we want to transmit in the discharge report among all the nurses who have seen the patient” [P2].

All of them agreed with the perception of a lack of time to complete, write and review the NDP.

“We write like robots...normally we write fast because we have little time.” [P1].

### Nurses’ perception of the contents

This theme was further divided into the following two subthemes:

a) The patient’s evolution during the hospital stay.

The nurses agreed that the information on medical diagnoses, interventions, treatments, the evolution of the patient and additional medical tests were reported very clearly.

“… diagnoses, treatments, interventions, this is very clear.” [P5].

However, hospital nurses acknowledged difficulties in providing nursing diagnoses.

“sometimes it is difficult to translate the information into nursing diagnoses” [P2].

Additionally, primary care nurses identified a lack of information regarding the education received and the empowerment achieved by the patient during the hospital stay.

“I have difficulties in finding out what has been explained to the patient.” [P2].

“The nurse at the hospital has given a lot of information to the patient but that’s all there is to it, I don’t know what has been explained to him or her.” [P5].

One nurse manager pointed out the importance of the patient’s pre-hospitalization conditions during hospital admission, which was identified as key information.

“There are many important things about the patient’s care that nobody knows when he or she is admitted.” [P8].

b) Nurses’ assessment of patients at hospital discharge.

In general, the participants perceived that they received adequate information about the nurses’ assessment of patients. However, primary care and nursing home nurses often found that important information for the continuity of care was not given.

Primary care nurses perceived poor information about the psychological and social aspects (family, social support, adaptation of the household, economic resources,) of the patient. “...sometimes when I arrive at a house it turns out that it doesn’t have an adapted toilet...” [P5].

With regards to this point, the hospital and management nurses explained that when necessary, the resources of the social workers were activated to work together in the preparation of the NDP.

“When nurses detect the need they can activate social services.” [P8].

They also considered that there was a need for reports to be individualized for the person who is being attended.

“It is not necessary to cover all the scales, each patient is different, the report should be adaptable and individualized.” [P5].

Primary care and nursing home nurses considered that the care plans proposed by the hospital nurses were sometimes not realistic. They considered that the frequency with which cures should be undertaken is often impossible given the workload that the nurses have and that the materials needed for some of the cures were not always accessible to primary care and nursing home nurses.

“Sometimes home cures are prescribed every 12 hours and this is normally practically impossible.” [P5].

This fact was identified as a generator of conflicts with patients, as they often perceived discontinuity in their care.

“… then patients demand that they receive this care and if we can’t give it to them they get angry” [P4].

All the participants agreed that the ideal report is one that is capable of conveying the same information as that provided by liaison nurses over the phone. Phone call were viewed as having the ideal information content.

“I would like to find the same information when reading the NDP as I receive when the liaison nurse calls.” [P4].

However, there was no consensus as to what the best patient assessment nursing model is and how this information should be conveyed in the report. One nurse manager referred to the importance of nurses from different care settings participating in the drafting of NDPs.

“...it would have been better if all the nurses, both those from the hospital and primary care, as well as those from the nursing homes, had participated in drafting the NDP.” [P8].

Finally, all the participants agreed that the information about forthcoming controls is a fundamental part of the NDP. Sometimes patients do not catch all the information given at hospital discharge and so having this content in the report is essential for ensuring continuity of care.

“It is also important that we can check that they know the day they need to return to the hospital for a check-up.” [P5].

### Nurses’ perception of higher priority patients in need of discharge planning

**Fig. 2:**
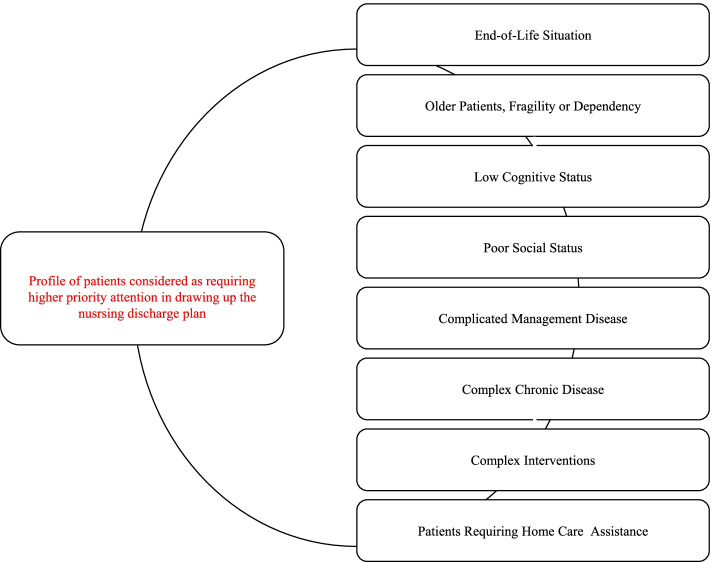
Profile of patients considered as requiring higher priority attention in drawing up the nursing discharge plan

Despite nurses agreeing that it was important to communicate information about each of the patients to other care settings through the NDP, they considered that there was a particular profile of patients that should be given special priority (Fig. 2).

“There are patients who need more continuity of care than others...it is important to identify these.” [P5].

… for example those with complex chronic conditions, or who have undergone complicated surgery.” [P1].

“I think end-of-life situations are the most vulnerable, we must guarantee the continuity of care in these patients … it’s very important for family members too” [P4].

They also believed that it was important to ensure that the primary care nurses’ first contact with these patients should take place as soon as possible. In this respect, primary care nurses clarified that it was not always necessary to go to the patient’s home, but that the first contact must be made by whatever means at an early stage.

“It is normally not necessary to visit the patient’s home immediately, but it is important to call patients once they arrive home after discharge at least.” [P8].

## Discussion

The main results of this study are that the nurses who participated in this study showed a high level of satisfaction with the NDP, especially those who work in nursing homes. It was considered that the NDP should have both a standardized structure and be flexible. Poor information about nursing discharge diagnoses, social and psychological aspects as well as a lack of information about the knowledge acquired by patients during their period of hospitalization are considered deficits of the current NDP.

The greater satisfaction of the nursing home nurses with NDP can be explained by the long-established cooperative work between this setting and the continuity of care units of the hospitals. On the other hand, primary care nurses had the lowest level of satisfaction with the NDP. According to Lu et al. [36], primary care professionals feel a lower level of involvement in the discharge planning process. In this respect, the results of the study suggest that we need to standardize and adapt care plans for nurses of different health care settings in order to ensure continuity of care. Similar results can be found in other studies, which show a need to communicate the information through the NDP in a way that is appropriate for each care level [20]. It has also been demonstrated that interprofessional collaboration increases the quality of care and patient safety [37]. In order to maintain and support continuity of care through the NDP, there is a need to improve the networking structures between nurses from different care settings.

The present study also highlighted the importance that the NDP should have a structure that was not only standardized but also flexible. While a standardized structure ensures the homogeneity of the information, greater flexibility in the structure would favor the quality and veracity of the content. These results are in line with the National Guidelines for On-Screen Presentation of Discharge Summaries [14] and may help in a better NDP, which is a key element in the discharge process [38]. Initial efforts to improve the quality of the NDP should focus on ensuring that a complete summary of the patient’s condition and circumstances is provided that is useful for all nurses in different settings. Given this, professionals from these different settings should participate together in the design of this new document.

With regards to compliance, the results of the present study show that the review and use of the completed NDP is widespread and performed systematically, which, according to Bradley & Mott [8], may have a positive impact not only on the continuity of care but also on the health of the professionals. However, the qualitative results of the study take a deeper look at correct compliance.

Firstly, hospital nurses often filled them out without contrasting the information with the patient and using abbreviations. Secondly, recognizable, commonly employed nursing language was not always used, leading to some information being misunderstood. This reaffirms the finding that providing a structured document with appropriate language is crucial to providing reliable and valid nursing data [39].

The assessment of the contents of the NDP highlighted a particular focus on patient evolution during the hospital stay, the assessment of the patient at discharge, and the continuity of care. With regards to the patients’ own role in their care, the nurses referred to a lack of information about what had been explained and taught to the patients about their illness and their empowerment to cope with it. A similar point was made by Blake et al. [40], who described the difficulties in reporting what patients had been taught during hospitalization through the report. The results of the present study also suggest that the identification of relevant information from primary care and nursing home nurses about patients’ competencies with regards to their illnesses before hospitalization can be useful.

Moreover, the nurses found that the NDP provided sufficient information about a person’s physical assessment. However, in both the quantitative and qualitative studies, they perceived a lack of information related to the psychological and social assessment of the patients. Kollbrunner [41] demonstrates how important social service workers are in providing information to patients about different discharge options. In this respect, nurses explained that when appropriate social workers were consulted to work together in the preparation of the NDP.

Our study also showed that the greater the level of professional experience, the greater the tendency to review the NDP, making clear the need to establish strategies to encourage younger nurses to review this document. Furthermore, the lack of postgraduate training was also found to be related to a lower perception of time to fill out the NDP. Therefore, nursing training related to the transfer of information must be viewed as a guideline for the continuity of care [42]. Receiving training in the best practices in health recording increases the satisfaction of healthcare workers and exponentially decreases the chance of the usability of these records being compromised [21].

### Limitations

There are some limitations to the findings of the present study. Firstly, the NDP assessment used in the hospitals of our health area may not be representative of NDPs used in other health systems. Secondly, with regards to quantitative research, the cross-sectional design, limits the study to the analysis of the relationships between variables without it being possibility to establish causality. Another limitation is the use of an ad hoc questionnaire. However, the values of Cronbach’s alpha indicate that the reliability was good. It should be noted that the use of a single focus group could limit the transferability of the results. However, the aim of using a focus group was to take an in depth look at certain aspects of the quantitative research results and by using an intentional sample we sought to ensure as broad a perspective on the NDP as possible.

## Conclusions

The current study assesses the perceptions of nurses from different healthcare settings about an NDP in terms of overall satisfaction, structure, completion, and content. Although in general nurses were satisfied with the NDP, some weaknesses did surface. While a standardized structure ensures the homogeneity of the information, greater flexibility in the structure would favor the quality and veracity of the content.

To maintain and support continuity of care through the NDP, there is a need to provide better networking structures among the nurses from different care settings. It is also necessary to reach an agreement on the structure and content of the NDP among the nurses from the different healthcare settings: providing sufficient time, using uniform nursing language, and adopting standardized care plans across care settings are key aspects of this. New strategies are needed to involve all nurses who have cared for the patient in a particular setting in filling out the NDP. This study highlights the essential contents of the NDP that are included, but more information about social and psychological situations together with information about the level of patient empowerment at hospital discharge is required. Finally, this study identifies patient profiles that have a greater need of discharge planning. These include patients who are older, are in end-of-life situations, have a low cognitive status or functional capacity, have poor social status, or have undergone complex surgery.

## Supplementary Information

**Additional file 1: Annex 1.** Questionnaire.

## Data Availability

Any additional data not presented in the present manuscript are available on demand from the corresponding author.

## Abbreviation

NDP: Nursing discharge plan
