# Supplementary material for: Evaluation and perceptions of a nursing discharge plan among nurses from different healthcare settings in Spain
Source: BMC Health Serv Res. 2022 May 28;22:710. doi: 10.1186/s12913-022-08109-9 (PMC9145205; doi:10.1186/s12913-022-08109-9)
Supplement: Supplementary file 1 — Additional file 1: Annex 1. Questionnaire. [file 12913_2022_8109_MOESM1_ESM.docx]

Annex 1. Questionnaire:

| Sociodemographic and working variables |
| --- |
| 1. Age |
| 2. Sex |
| Woman Man |
| 3. Years after completing university studies |
| 4. Years of work experience in the same care level |
| 5. Employment relationship |
| Permanent Temporary |
| 6. Care setting |
| Primary healthcare Hospital care Nursing home |
| 6. Function |
| Care Management Liaison nurse Case management nurse |
| 7. Training |
| Continuous Postgraduate |
| 8. Have you published in the last 5 years? |
| Yes No No answer |
| 9. Have you attended congresses or activities related to the continuity of care? |
| Yes No No answer |
| **Perception of Girona’s Nursing Discharge Report** |
| 10. What is your overall satisfaction with the report?  1.Extremely satisfied 2.Very satisfied 3.Moderately satisfied 4.Slightly satisfied 5.Not at all satisfied |
| 11. Do you review and consider the reports?  1. Always 2. Often 3. Sometimes 4. Rarely 5. Never |
| 12. Do you think the time available to perform or review the reports is adequate?  1.Strongly agree 2. Agree 3.Neither agree nor disagree 4. Disagree 5. Strongly disagree |
| 13. Please score the quality of the information provided in the report for each of the following items?  (from 1 to 10, where 1 is the poorest quality of information and 10 the best)  Medical diagnoses at discharge:  Nursing diagnoses at discharge: _____  Summary of the hospital admission: _____  Physical assessment at discharge:_____  Social assessment at discharge: _____  Care plan: _____  Recommendations at discharge: _____  Forthcoming controls: ____ |
